# The Effects of the Re-imposition of US Sanctions on Food Security in Iran

**DOI:** 10.34172/ijhpm.2020.207

**Published:** 2020-11-02

**Authors:** Jalal Hejazi, Sara Emamgholipour

**Affiliations:** ^1^Department of Nutrition, School of Medicine, Zanjan University of Medical Sciences, Zanjan, Iran.; ^2^Department of Management & Health Economics, School of Public Health, Tehran University of Medical Sciences, Tehran, Iran.

**Keywords:** Sanctions, Iran, Food Security, Consumer Price Index

## Abstract

**Background:** Following the re-imposition of US sanctions against Iran in 2018, unprecedented inflation has occurred in Iran’s food market that will undoubtedly affect the food security of the Iranian people. The present study aims to determine the effects of the sanctions on food prices and food security of Iranian households.

**Methods:** Interrupted time series (ITS) analysis was applied to assess the effects of sanctions on the average retail price of food products in Iran. Household food security was estimated by calculating the share of household food expenditure. Costs of following a healthy diet based on the food pyramid were estimated.

**Results: **The import dependency ratio of Iran’s food market was about 25%. After sanctions due to the limitations in international financial exchanges a significant increase in the prices of all food groups occurred in 2018, the year after the re-imposition of sanctions. The highest inflation rate was observed in vegetable, meat, and fruit groups. The percentage of urban and rural households in Iran that were prone to food insecurity increased from 8.84% and 25.17% to 11.2% and 29.2%, respectively, from 2017 to 2019. The annual average cost of a healthy diet for a sample Iranian family of 3.3, based on the current prices, is 341 866 008 IRR (US$2849) which is 3.6 times greater than the average amount Iranian families spent on food last year (94 505 000 IRR or US$788).

**Conclusion:** After the re-imposition of US sanctions against Iran, food insecurity as a result of economic vulnerability, has increased and due to the current status of food prices and incomes, following a healthy diet has become more difficult for most Iranians. This makes the Iranian population more prone to chronic diseases in the near future and if this trend persists, it places the country in danger of food crisis and political instability.

## Background

**Key Messages**

** Implications for policy makers**

- A significant increase in the prices of all food groups has occurred during the year following the sanctions.
- The highest inflation rate was observed in vegetable, meat, and fruit groups.
- Following a healthy diet is nearly impossible for most Iranians due to the current food prices and income levels.
- Ordinary Iranian citizens, especially those in vulnerable groups such as children, are the main victims of the sanctions.
- Immediate food assistance programs are needed for vulnerable groups by the Iranian government.

**Implications for the public**

After the re-enactment of US sanctions against Iran in 2018, severe inflation has occurred in Iran, especially in the food market. This inflation was even more remarkable in nutritious foods such as vegetables, fruits, meat, etc. Foods with less nutritional value and high-calorie density such as starchy foods and oils are cheaper and the government pays a subsidy for some of them (white bread for instance). Therefore, low-income families tend to gain most of their daily calorie intake from these foods. These unhealthy foods may put the families at risk of obesity and chronic diseases such as diabetes and cardiovascular diseases. The results of this study showed that most of the Iranian families were unable to follow an average healthy diet because of their current income and expenses. Creating efficient food assistance programs by government and the international community, founding food banks with the assistance of charities and non-governmental organizations, and participation of individuals in nutritional education programs and learning how to plan a cheap and balanced diet may help Iranians to reduce the unfavorable effects of sanctions on their health and nutrition status.

As a result of the withdrawal of the US Government’s Trump administration from the Iran nuclear deal, and the re-enactments of US sanctions against Iran in 2018, Iran’s currency (the rial) collapsed, losing two-thirds of its value, giving rise to unprecedented inflation in the food market.

 Although US officials have stated that food and medications are not subject to sanctions against Iran, due to the problems that sanctions have created for trade and transfers of goods, sale of oil, and international financial exchanges of Iran, the sharp rise in the exchange rate has pushed up the prices of imported goods such as food and has led to imported inflation. Furthermore, as imported goods have become more expensive, demand for domestic food products has increased, but due to the increase in imported input prices, the supply of domestic food products that use these inputs has not been able to meet the demands.

 The Food and Agriculture Organization (FAO) defines food security as “A situation that exists when all people, at all times, have physical, social and economic access to sufficient, safe and nutritious food which meets their dietary needs and food preferences for an active and healthy life.”^1^ The inverse association between food prices and food security, especially in developing countries, is well established by several studies.^2,3^ It was estimated by FAO that after the 2008 food price crisis and the global rise in cereal prices, an additional 40 million people were pushed into hunger.^4^

 Different methods have been introduced for screening for food security and measuring it. Food expenditure surveys are one of the most preferred methods that can provide more comprehensive and reliable information in this regard.^5^ In a manual for measuring food security using the household expenditure survey, Smith and Subandorointroduced economic vulnerability as an indicator of food security.^6^ They defined economic vulnerability as “The percentage of total household expenditures devoted to food over the reference period.”^6^ This method of measuring food insecurity is based on the fact that economically vulnerable families have to sacrifice the quantity and quality of their consumed food to reduce their expenses.

 Food prices can also affect diet quality.^7^ The indispensable requirements for having a healthy diet are nutrient adequacy, moderation, and variety.^8^ There is accumulating evidence that dietary diversity and quality can be negatively affected by food price rises, particularly among the poorest.

 The present study aimed to evaluate the impacts that US sanctions against Iran on food prices, food security (based on economic vulnerability method), and on the dietary quality of Iranian households.

## Methods

###  Study Design

 First, the share of imports in food consumption and food self-sufficiency ratio was calculated to confirm that food items are subject to imported inflation. Then, the impact of sanctions on the prices of the major food groups was assessed. For this purpose, the average prices of 6 food groups during 27 consecutive months, from April 2017 till June 2019, were assessed, and interrupted time series (ITS) regression was applied to analyze the immediate and gradual effects. Subsequently, the number of Iranian households who are facing low, modest, and severe food insecurity during the period from 2017 to 2019 was obtained, and eventually, the number of Iranians who can follow a healthy diet was estimated.

###  Data Sources

 Statistics of production, import, and export of agricultural products and foodstuffs were obtained from the Statistical Center of Iran (SCI). Average retail prices in urban areas from April 2017 to June 2019 were also gathered from the SCI.^7^ The food prices in May 2019 were used to appraise the cost of following a healthy diet based on the food pyramid in Iranian households. Concerning staple foods of Iranian cuisine such as bread or pasta which were not included in the report list of SCI, average prices reported by economic news agencies in those time frames were applied.

 To estimate the number of the Iranian households that are facing food insecurity, the average annual household income and expenditure including non-food expenditures (including housing, transport and communication, medical care and health services, clothing and footwear, etc) and food expenditures were obtained from the statistical data of the “Iranian Urban and Rural Household Income and Expenditure Survey of the Year *1397* (March 20, 2018 - March 20, 2019).”^9^

 In the present study, a healthy diet was defined according to guidelines specified in “Food-Based Dietary Guidelines for Iran” published by the Iranian Ministry of Health in 2015.^10^ Except for only a few Iranian foods included in some food groups, these guidelines are precisely in line with United States Department of Agriculture’s “Food Pyramid.” The pyramid shows the optimal range of servings for each food group. The number of appropriate servings for any individual depends on one’s energy requirement, which in turn is conditioned by age, sex, body size, activity levels, etc.^11^ However, based on “Good Food Basket for the Iranian Community” which is published by the Iranian Ministry of Health, the average energy requirement of Iranians is 2381 kcal.^12^ In keeping with this, the most favorable number of servings for each food group and the serving size of each are shown in Table 1. Since the total number of servings of fat is not mentioned in the pyramid and fat dietary reference intake for healthy adults is between 20%-35% of total energy expenditure,^13^ we calculated 25% of calories as fat which equals 66 g.

**Table 1:** Appropriate Number of Servings and the Serving Size of Each Food Group for a 2381 Kcal Diet

**Food Groups**	**Number of Servings**	**Serving Size (g)**
Cereals	10	Bread, rice (raw), pasta (raw): 40
Vegetables	4	Cucumber, tomato, potato, onion: 75
Fruits	4	Banana, apple, orange: 150
Dairy products	3	Milk: 250, yogurt: 200, cheese: 40
Meat group	3	Lamb, beef, chicken (raw): 100, egg: 120, pinto beans, lentils (raw): 40

###  Data Analysis

 The ratio of imported food and agricultural products to the added value of the agricultural sector was calculated for the period from 2011 to 2018 to show that to what extent Iran’s food market is dependent on imports.

 The ITS model was applied to assess the impact of the re-imposition of US sanctions against Iran concerning food prices. ITS model is explained in detail elsewhere,^14,15^ but in brief, we used equation 1 to assess the immediate and gradual effects of these sanctions.

Yt = B0 + B1*time + B2*intervention + B3*time after intervention ^14^Eq.(1)

 In this equation, *Yt* is the Consumer Price Index of each food group in a month, time is a continuous variable indicating the number of the months that have elapsed since the start of the observation (April 2017), and intervention is an indicator variable for time *t* occurring before (0) or after (1) the sanctions. *β0* represents the intercept or starting level of the outcome variable, and *β1* is the slope or trajectory of the outcome variable until the introduction of the intervention. *β2* represents the change in the level of the outcome that occurs in the period immediately following the introduction of the intervention (compared to the counterfactual), and *β3* represents the difference between pre- and post-intervention slopes of the outcome. The sum of *β1* and *β3* is the post-intervention slope. Analyses were conducted using EViews 10.

 The total price of one serving of each foodstuff was calculated (based on the data presented in Table 1 and the average retail prices of 1 kg of some foodstuffs during May 2019 published by the SCI). Afterward, the average prices of one serving of each 5 food groups (cereals, vegetables, fruits, dairy products, and meats) were computed. For instance, the total average prices of one serving of beef, lamb, poultry, egg, pinto beans, and lentils were calculated. Next, these values were multiplied by the corresponding number of servings for each food group (Table 1) and were summed to achieve the average cost of a healthy diet for one person.

 According to the SCI, the average family size in urban areas of Iran is 3.3.^16^ Therefore, the daily food cost of a family of 3.3 was calculated and was later multiplied by 365 to obtain the average cost of a healthy diet for a sample Iranian family in one year. Following this, the resultant value was compared with the budget that Iranians can spend on food.

 To estimate the population proportion with food insecurity, we calculated the economic vulnerability of that population, based on the instructions of the International Food Policy Research Institute.^6^ Economic vulnerability is defined based on the share of food expenditure.

*Share of food expenditure (%) = household food expenditure/household income* ^6^

 Classification of economic vulnerability to food security based on Smith and Subandoro’s manual is presented in Table 2.

**Table 2:** Guideline for Classification of Economic Vulnerability to Food Insecurity

**Economic Vulnerability**	**Percentage of Expenditures on Food**
Very high	+ 75%
High	65%-75%
Medium	50%-65%
Low	<50%

Source: Smith and Subandoro.

## Results

 The calculated average ratio of imported food and agricultural products to the added value of the agricultural sector was 25% from 2011 to 2018 (data is not shown). Also based on the World Food Program report, Iran’s food market has some dependency on imports. They reported food self-sufficiency of 53%-82% during different years after 2000.^17^

 The average retail prices of 1 kg of some main foodstuffs in May 2017, May 2018 (at the time of the re-enactments of the US sanctions against Iran), and May 2019 are listed in Table 3. As it is shown, 8 of the food items had an inflation rate of more than 100% after sanctions, whereas 4 of these food items had even negative inflation rates during the year before sanctions.

**Table 3:** Average Retail Prices of 1 kg of Some Foodstuffs

**Food Item**	**Price of 1 kg [IRI Rials (USD)** ^a^ **] (May 2017)**	**Price of 1 kg [IRI Rials (USD)** ^b^ **] (May 2018)**	**Price of 1 kg [IRI Rials (USD)** ^c^ **] (May 2019)**	**Percentage of Change 2017-2018**	**Percentage of Change 2018-2019**
Rice (Iranian)	126 440 (3.38)	136 657 (2.24)	206 097 (1.57)	8.08	50.81
Lamb	366 477 (9.64)	447 167 (7.33)	992 218 (7.57)	22.02	121.89
Beef	349 531 (9.20)	405 413 (6.64)	931 732 (7.11)	15.99	129.82
Chicken	67 796 (1.78)	78 856 (1.29)	118 899 (0.91)	16.31	50.78
Milk	26 735 (0.70)	28 522 (0.47)	47 853 (0.36)	6.68	67.78
Yogurt (pasteurized)	36 580 (0.96)	39 594 (0.65)	60 625 (0.46)	8.24	53.12
Cheese (Iranian)	57 856 (1.52)	59 669 (0.98)	89 904 (0.69)	3.13	50.67
Egg	53 483 (1.41)	83 195 (1.36)	89 255 (0.68)	55.55	7.28
Butter	28 489 (0.75)	34 044 (0.56)	45 596 (0.35)	19.50	33.93
Liquid vegetable oil	53 698 (1.41)	54 457 (0.89)	80 185 (0.61)	1.41	47.25
Banana	51 977 (1.37)	71 120 (1.16)	141 352 (1.08)	36.83	98.75
Apple	54 558 (1.44)	43 301 (0.71)	105 072 (0.80)	-20.63	142.66
Orange	51 389 (1.35)	45 353 (0.74)	84 346 (0.64)	-11.74	85.98
Cucumber	23 337 (0.61)	25 673 (0.42)	55 681 (0.42)	10.01	116.88
Tomato	37 937 (1.00)	19 455 (0.32)	55 797 (0.42)	-48.72	186.81
Potato	21 355 (0.56)	17160 (0.28)	58 569 (0.45)	-19.65	241.32
Onion	18 950 (0.50)	18 176 (0.30)	73 802 (0.56)	-4.08	306.04
Pinto beans	114 247 (3.01)	119 443 (1.96)	155 334 (1.18)	4.55	30.05
Lentils	85 308 (2.24)	79 789 (1.31)	94 825 (0.72)	-6.47	18.85
Sugar	33 082 (0.87)	33 372 (0.55)	77 489 (0.59)	0.88	132.20

^a^The exchange rate in May 2017 was 1 USD ≈ 38 000 IRR in Iranian exchanges. ^b^The exchange rate in May 2018 was 1 USD ≈ 61 000 IRR in Iranian exchanges. ^c^The exchange rate in May 2019 was 1 USD ≈ 131 000 IRR in Iranian exchanges.

 Figure shows the price trend of different food groups from April 2017 to June 2019. As it is presented, prices for all of the food groups have increased dramatically after the re-imposition of the sanctions and the sharpest slope belongs to the vegetable, meat, and fruit groups.

**Figure:**
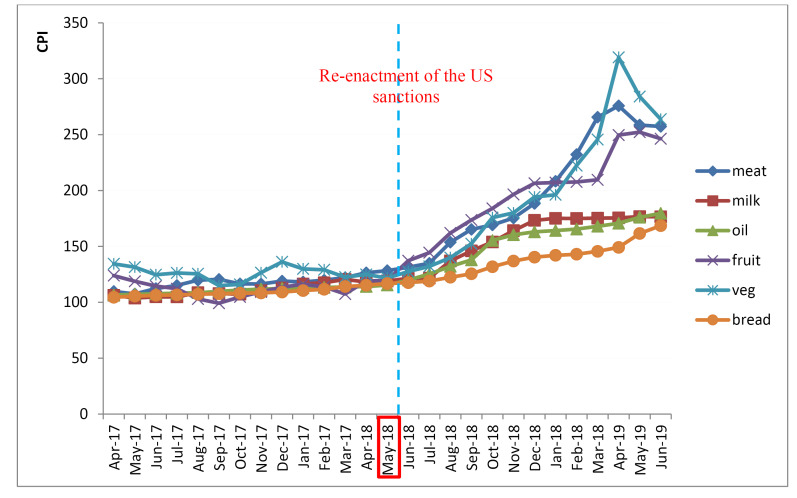
Consumer Price Index Trend of Different Food Groups between April 2017 to June 2019. Abbreviation: CPI, Consumer Price Index.

 The results of the ITS analysis are summarized in Table 4. While prices of all food groups were slightly reduced immediately after the re-enactment of US sanctions (cereals [-0.28], vegetables [-1.102], fruits [-0.58], dairy products [-0.28], meats [-0.76], and oils [-0.34]), the price growth rate of all food groups and their price index increased at a rate faster than the previous trend.

**Table 4:** Immediate and Gradual Effects of the Re-Enactment of US Sanctions against Iran, affecting the Prices of Different Food Groups: The Results of ITS Analysis

	**Intercept β0**	***P***	**Time Effect β1**	***P***	**Intervention Effect β2** **Immediate Effect (SE)**	***P***	**Time** ^a^ ** Intervention β3 Gradual Effect (SE)**	***P***	**The Trend of CPI After Sanction β1+ β3**
LOG*(MEAT)	4.69 (0.027)	.00	0.009 (0.003)	.00	-0.76 (0.07)	.01	0.054 (0.004)	.00	.063
LOG(MILK)	4.62 (0.028)	.00	0.012 (0.003)	.00	-.03 (0.071)	.00	0.021 (0.004)	.00	.033
LOG(OIL)	4.66 (0.020)	.00	0.006 (0.002)	.02	-0.34 (0.051)	.00	0.027 (0.003)	.00	.033
LOG(VEGEtables)	4.84 (0.040)	.00	-0.001 (0.004)	.72	-1.10 (0.090)	.00	0.070 (0.006)	.00	.069
LOG(FRUIT)	4.73 (0.030)	.00	-0.002 (0.004)	.66	-0.58 (0.090)	.00	0.055 (0.006)	.00	053
LOG(BREAD)	4.63 (0.009)	.00	0.007 (0.001)	.00	-0.28 (0.025)	.00	0.019 (0.001)	.00	.026

Abbreviations: CPI, Consumer Price Index; ITS, interrupted time series; SE, standard error. * The logarithmic form of variables was used to indicate the percentage of change for each dependent variable.

Table 5 shows the percentage of urban and rural households in Iran that were prone to food insecurity based on the share of food expenditure. As it is shown, in 2017, 8.84% of urban families and 25.17% of rural families had some degrees of food insecurity whereas in 2019 these values increased to 11.24% and 29.2% for urban and rural households, respectively; which shows an increasing trend in the food insecurity of both urban and rural families.

**Table 5:** Percentage of Urban and Rural Household in Iran Prone to Food Insecurity From 2017 to 2019

	**Urban Households**				**Rural Households**			
	**Very High **	**High **	**Medium **	**Low **	**Very High **	**High **	**Medium **	**Low **
2017	1.91	1.54	5.39	91.16	8.37	4.40	12.40	74.83
2018	2.83	1.76	6.00	89.41	10.09	4.46	13.25	72.20
2019	3.40	1.84	6.00	88.76	10.68	5.21	13.31	70.80

Table 6 shows the average price of a healthy diet based on the food pyramid for one person and a sample family, the annual cost of each food group, and the average cost of a healthy diet for an Iranian family. As is shown, the average annual cost of a healthy diet for a sample Iranian family is 341 866 008 rials (US$2848.89).

**Table 6:** The Average Price of a Healthy Diet for 1 Person and a Sample Family

**Food Groups**	**The Daily Cost for 1 Person **		**The Daily Cost for a Family of 3.3**		**The Annual Cost for a Family of 3.3**	
	**Rials**	**USD**	**Rials**	**USD**	**Rials**	**USD**
Cereals	37 612	0.31	124 119.6	1.03	45 303 654.0	377.53
Vegetables	18 289	0.15	60 353.7	0.50	22 029 100.5	183.58
Fruits	66 155	0.55	218 311.5	1.82	79 683 697.5	664.03
Dairy products	31 280	0.26	103 224.0	0.86	37 676 760.0	313.97
Meat group	112 501	0.94	371 253.3	3.09	135 507 454.5	1129.23
Fats	17 987	0.15	59 357.1	0.49	21 665 341.5	180.54
Total	283 824	2.36	936 619.2	7.81	341 866 008.0	2848.89

## Discussion

 To the best of our knowledge, this is the first study that has investigated the effects of US sanctions against Iran on food prices, food security, and the dietary quality of Iranian households. According to our analysis, the prices of most food items increased by more than 50% during the year after the sanctions. The highest inflation rates were observed in nutritious foods (such as fruits, vegetables, and meat products), and the least nutritious foods (such as white bread and oils), had the lowest inflation rates. Food insecurity as a result of economic vulnerability increased dramatically between the years 2017 and 2019, in both rural and urban households.

 Based on the current prices, the average cost of a healthy diet for a sample Iranian family is 341 866 008 rials (US$2848.89) per year which is 3.6 times greater than the amount that an average Iranian family could spend on food and tobacco last year (US$94 505 000 rials or US$787.5). This amount is 9.5 and 2 times higher than the food and tobacco expenditure of the lowest and highest decile of households, respectively. This amount consumes nearly 80% of the total income of an average Iranian family.

 To establish the average cost of a healthy diet for an Iranian family, it was assumed that all of the Iranian families consume homemade foods. That being said, the results of the previous studies demonstrate that this amount can rise to 40% for the families that eat most of their meals out.^18^

 It is worth mentioning that the food market was not the only market that went through this sharp inflation. According to the SCI, the point-to-point inflation rate rose to 52.1% in May 2019, compared with May 2018.^19^ Regarding some goods and services including housing, this rate was even higher.^19^ This means that the Iranians’ ability to spend money on foods will irrefutably plummet to a greater extent this year.

 In this study, we calculated the average price of a healthy diet. It is possible to devise a more economical plan for having a healthy diet; however, regarding the current precarious food purchasing power of the Iranians, even if we could reduce the cost of the healthy diet by half, most of the Iranians still would not be able to follow a healthy diet.

 In a systematic review by Kokabisaghi, it was concluded that due to the limitations that were imposed on trade, banking, the financial system, and shipments as a result of sanctions, transferring any goods to Iran, including food and medications had become extremely difficult and expensive.^20^ Based on a report, these limitations have increased the cost of trading by 40%.^21^ In 2012 and after the first round of nuclear sanctions against Iran, the United Nations Children’s Fund (UNICEF) described Iran as a country under tight unilateral economic sanctions that adversely affect the environment, public health, and the socio-economic determinants of health of ordinary people, especially children.^22^

 Based on a systematic review by Mohammadi-Nasrabadi et al, there are only a few previous studies that have assessed the food insecurity in Iran based on household income/expenditure and they have reported that prevalence of food insecurity in both rural and urban areas of Iran is 10%.^5^A meta-analysis of the studies, which used dietary-recall method for assessing food insecurity, showed non-significant alterations in the prevalence of mild (from 8.8% to 9.3%), moderate (from 5.4% to 5.6%) and severe (from 3.8 to 3.7) food insecurity from 1994 to 2004.^5^ However, in the present study, during the period between 2017 and 2019, percentage of severe (very high + high) food insecurity was increased by more than 50% and 24% in urban and rural areas, respectively; which is an indication of the effects of US sanctions on the food security of Iranians.

 It is well established that a healthy diet costs more than an unhealthy one and nutrient-rich foods are costlier than foods containing empty calories.^23^ It is shown in the present study that the most expensive food groups of a healthy diet are meats and fruits, while the least costly one is the fat group. As a result, the current economic situation will impel the Iranians to consume energy-dense foods such as fats, sugars, and refined grains (this is mainly because the government pays a subsidy for some foods like white bread). Thus, as a result of following this unhealthy diet, an unprecedented breakout of obesity and its related diseases such as cardiovascular diseases, diabetes, and cancers will be inevitable in the near future.^23,24^ Moreover, if the current inflation rate in the food market persists, it is predictable that the on-going hidden hunger will turn into a real food crisis.

 There are very few studies on the impact that the sanctions by the United Nations (UN) or the US have made on the food security of citizens of sanctioned countries; however, studies from Iraq^25,26^ and Cuba^27^ can confirm the unfavorable effects of sanctions on the food security of their population. Furthermore, there are numerous studies that testify to the devastating effects of sanctions on human rights and different aspects of health such as the availability of medications or medical devices for ordinary people.^20,28-30^

 After the re-enactment of US nuclear sanctions, Iran’s government adopted some policies to reduce the devastating effects of the sanctions on ordinary people. In April 2018, the Central Bank of Iran allocated a subsidized rate of 42 000 Iranian rials per US dollar to Iranian companies importing designated “essential goods” (the dollar is being exchanged for more than 120 000 rials on the open market in Iran). Some food items including various kind of meats, legumes, vegetable oils, rice, etc were listed as essential goods; however, by observing the prices of these items during the year after the re-imposition of sanctions, it can be concluded that the initiative taken by Iran Central Bank had minimal, if any, effects in controlling the food prices, and the fact that the average price of meat nearly tripled during this period testifies to the truth of this claim. Thus, assessing the efficiency of this policy seems to be necessary for the Iranian government. Creating efficient food assistance programs, founding food banks with the assistance of charities and non-governmental organizations, and then implementing nutrition education programs to teach people about how to have the right food choices according to their budget are some measures that Iran’s government can take to reduce unfavorable impacts of the sanctions on Iranian households.^31,32^

 Even before US sanctions against Iran, this country was considered as a high-risk country in terms of food security.^33^ However, at present, the situation has aggravated and if this unfavorable condition persists, Iran will be on the verge of a food crisis. This state of affairs prevails while the UN is following the Zero Hunger Challenge; an effort to put an end to hunger all over the world before 2030.^34^ The continuation of the present situation will result in the joining of millions of Iranians to the undernourished population in the years to come. Therefore, it is urgent that the international institutions such as the UN take action against this issue. It is quite clear that like many existing conflicts in the world, the only victims of the current political conflicts between the United States and Iran are the ordinary citizens of Iran, especially the ones in the vulnerable groups such as the children and the elderly.

 The results of the present study have some limitations. Based on the nature of this study it was not possible to have a control group. As a result, we were not able to control the effects of possible confounding variables such as weather, agricultural production, etc on the food security of Iranians on this time frame.

## Conclusion

 After the re-imposition of US sanctions against Iran, unprecedented inflation occurred in Iran’s food market, and due to the current high prices of foodstuff and Iranians’ low income, following an average healthy diet is nearly impossible for most of the population. This is likely to make the Iranian people more prone to chronic diseases in the near future and if this trend persists, it can put the country in danger of a food crisis and political instability.

## Ethical issues

 This study is a secondary analysis of data from public reports of Statistical Center of Iran and as such, no ethical approval was required.

## Competing interests

 Authors declare that they have no competing interests.

## Authors’ contributions

 JH contributed substantially to the conception and design of the study, the acquisition of data, data analysis and wrote the paper. SH contributed substantially to the conception and design of the study, data analysis and provided critical revision of the article.

## Funding

 This research did not receive any specific grant from funding agencies in the public, commercial, or not-for-profit sectors.

## Authors’ affiliations

^1^Department of Nutrition, School of Medicine, Zanjan University of Medical Sciences, Zanjan, Iran. ^2^Department of Management & Health Economics, School of Public Health, Tehran University of Medical Sciences, Tehran, Iran.
